# A dynamic model of circadian rhythms in rodent tail skin temperature for comparison of drug effects

**DOI:** 10.1186/1740-3391-10-1

**Published:** 2012-01-05

**Authors:** Dorothee Girbig, Karsten Keller, Katja Prelle, Vladimir Patchev, Richardus Vonk, Bernd-Wolfgang Igl

**Affiliations:** 1 Bioinformatics Group, Max Planck Institute of Molecular Plant Physiology, Potsdam, Germany; 2 Institute of Mathematics, University of Lübeck, Germany; 3 Toxicology, Global Early Development, Bayer HealthCare, Wuppertal, Germany; 4 TRG Women's Healthcare, Global Drug Discovery, Bayer HealthCare, Berlin, Germany; 5 Global Drug Discovery Statistics and Experimental Medicine Statistics, Bayer HealthCare, Berlin, Germany

## Abstract

Menopause-associated thermoregulatory dysfunction can lead to symptoms such as hot flushes severely impairing quality of life of affected women. Treatment effects are often assessed by the ovariectomized rat model providing time series of tail skin temperature measurements in which circadian rhythms are a fundamental ingredient. In this work, a new statistical strategy is presented for analyzing such stochastic-dynamic data with the aim of detecting successful drugs in hot flush treatment. The circadian component is represented by a nonlinear dynamical system which is defined by the van der Pol equation and provides well-interpretable model parameters. Results regarding the statistical evaluation of these parameters are presented.

## Background

The rapid drop in hormone production during the climacteric implies a variety of changes in the physiology of the female body. This can lead to hot flushes which occur in around 70% of all women in Europe and North America [1]. Hot flushes are characterized by a sudden sensation of intense heat, accompanied by flushing of certain peripheral skin parts as well as intensive sweating. The pathophysiology of hot flushes is not yet completely understood, but their occurrence is assumed to originate in disturbances of the thermoregulatory processes in the hypothalamus, which acts as the body's thermostat [2].

**Figure 1:**
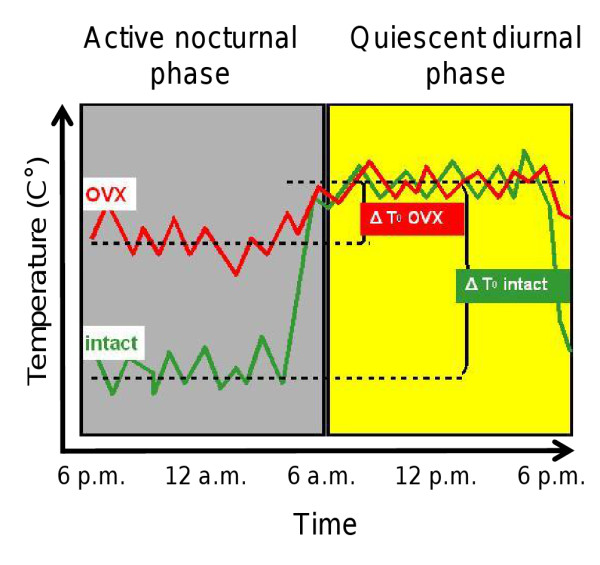
**Principles of the ovariectomized rat model**. The effects of ovariectomy (OVX) on circadian rhythms in the tail skin temperature of the rat. In the intact animal, the temperature baseline is lowered during the active nocturnal phase. In the ovariectomized animal, the temperature in this episode is elevated, which results in a reduction of the distance to the baseline of the quiescent phase. The graphics have been adapted from [3].

An animal model suitable for assessing the effectiveness of drug candidates in hot flush treatment is the ovariectomized rat model [3]. Here, a menopause-like state is simulated by ovary removal (ovariectomy, OVX), resulting in estrogen deprivation. It is assumed that the lack of estrogens lowers the set point for the activation of heat-dissipating mechanisms. As shown in Figure 1, this results in a lack of decrease in tail skin temperature during the rats' active phase at night and, consequently, flattening of the circadian oscillations in this parameter. Then, the effectiveness of a substance in compensating the effects of estrogen deprivation can be assessed by its capability to restore the amplitude of circadian rhythms in the tail skin temperature.

The ovariectomized rat model has been widely applied for the evaluation of substance effects for treating menopausal syndromes [4-8] and for investigating the underlying pathophysiology [9].

So far, there have been limited efforts in developing sophisticated techniques based on characteristic properties of the rhythms when evaluating the effects of a tested substance on circadian rhythms. Important characteristics are (1) a dynamic amplitude function monitoring the adaptation of oscillations to new treatments and (2) the capability of the circadian pacemaker to maintain limit cycles after sufficient adaptation to the treatment.

It should be noted that circadian rhythms in the body core have been subject to intensive research in the last few decades, and appropriate mathematical models have been formulated. These approaches comprise, for example, harmonic regression models based on weighted sums of multiple sines and cosines of varying frequencies [10], or differential equation models [11,12].

In this work, we present an approach for analyzing circadian rhythms in tail skin temperature, which is based on a nonlinear dynamical model defined by the van der Pol equation. Originally proposed for the analysis of circadian rhythms within body core temperature data [13], it was mainly applied for studying the human circadian clock and its reaction to external light stimuli [12]. It must be pointed out that, in addition, this model exhibits some properties making it well-suited for the investigation of the tail skin temperature of the rat. In particular, it describes sinusoidal oscillations in body temperature weighted by a continuous amplitude function, which allows monitoring of rhythm adaptations to a new treatment.

Here, the van der Pol model is fundamental for analyzing time-dependent amplitudes. The model parameters intuitively provide interpretable measures of the properties characterizing the oscillation. For example, they contain information about the start amplitude, the final amplitude at the end of a treatment period and about the individual pace of amplitude adaptation in each subject.

We apply the van der Pol equation to investigate the effects of the natural estrogen 17*β *Estradiol (*E2*) and of the synthetic hormonally active steroid *Tibolone *on circadian rhythms in the tail skin temperature. Both substances are known to reduce hot flushes in affected women successfully [14] and can thus be used as references to evaluate the capability of the proposed methods for detecting treatment effects.

## Methods

### The Experiments

**Figure 2:**
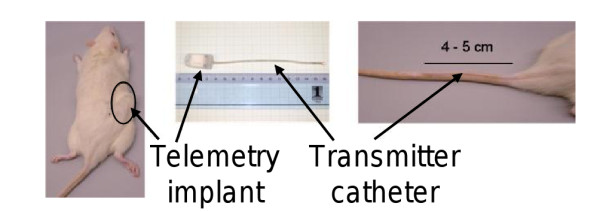
**Telemetric device**. Measurement device and transmitter used in the telemetry experiments (source: Bayer Pharma AG).

The effects of *E2 *and *Tibolone *were tested using the ovariectomized rat model. During ovariectomy, a temperature sensor was tunneled under the tail skin at a distance of 4 - 5 cm from the base of the tail, and a transmitter was implanted (see Figure 2 for details). Temperature values were averaged over 10 seconds for each record; records being taken every 3 minutes. A day-/night-rhythm was simulated by a 'daytime period' of 6 a.m. - 6 p.m. in which room light was switched on, and by a 'nighttime period' of the remaining 12 hours where the light was switched off. Each morning at 6 a.m., animal husbandry-related activities began. Treatment was administered between 8:30 - 9:30 a.m. each day. In the remaining time, animals were undisturbed in order to minimize the amount of perturbing influences on the measurements.

In contrast to the experiments described in literature [3-5,7,8], measurements were started directly after ovariectomy in order to prevent desensitization of estrogen receptors due to the OVX-induced deprivation of the cognate ligand.

**Figure 3:**
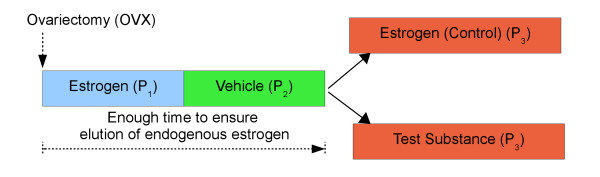
**Different phases and treatment groups**. Experimental protocol of the OVX experiments.

As depicted in Figure 3, the experiment was split up into three consecutive phases:

1. In the estrogen phase (P_1_), animals were treated with *E2 *for 6 days in order to simulate the physiological conditions prior to OVX. This allowed circadian rhythms to be maintained after the elimination of estrogen production. Their magnitude serves as a reference of the animals' original rhythms prior to OVX.

2. In order to ensure complete elimination of circulating *E2*, no medication was administered for 6 days (vehicle phase, P_2_). Nevertheless, a vehicle was applied each day to keep habituation to the experimental procedure. The aim of this phase was to guarantee that all effects observed in the subsequent test phase were evoked by the test substance.

3. For the treatment phase (P_3_), animals were randomized to a test group (treatment by *Tibolone*), and a control group (treatment by *E2*), each comprising 10 animals. In each group, treatment was administered for a period of 10 days.

### Mathematical Model

Circadian rhythms in body temperature can be studied by dynamic models based on the van der Pol equation [13]. So far, applications have mainly been focused on the analysis of measurements in the human body core [11,12]. In doing so, reactions of the circadian pacemaker in the hypothalamus to external light stimuli or changes in light-/dark-rhythms are investigated in order to get deeper insights about the underlying physiological processes. The application of the model to circadian rhythms in tail skin temperature requires some adjustments because of the different properties of human body core temperature and rat tail skin temperature and because of the different experimental protocols.

Measurements of body core temperature usually have far less variability than tail skin temperature recordings since the latter are strongly influenced by thermoregulatory-induced variations in vasodilatation. These can result in high frequency perturbations with peaks that often exceed the circadian amplitude making the intrinsic circadian component difficult to detect. Furthermore, tail skin temperature is more sensitive to changes in ambient temperature (evoked for example by rats sitting on their tails) than the well-tempered body core.

We assume that the observed time series ${\left(y_{n}\right)}_{n=0}^{N-1}$ of some length *N *is given by the temperature values

(1) $$y_{n}=x\left(n\Delta t\right)+c\left(n\Delta t\right)+e_{n},n=0,1,\ldots ,N-1,$$

with a sampling interval Δ*t *of 3 minutes. *y_n _*is the *n*-th temperature measurement, where we start with measurement 0. Furthermore, *c*(*t*) = *c*_0 _+ *c*_1 _· *t *with *c*_0 _*>*0 and *c*_1 _∈ ℝ describes a general baseline and a linear trend, while *e_n _*is a random error term for each *n*.

The circadian component *x*(*t*) needs to incorporate the most important properties of circadian rhythms in the living organism, which are (1) the capability to be sustained without external stimuli and (2) a dynamic amplitude function to monitor the adaptation of the extent of circadian oscillation to different treatment modes in different experimental phases. Similar to the circadian pacemaker maintaining steady oscillations in the undisturbed organism, the model should incorporate oscillations approaching a constant amplitude.

The van der Pol equation

(2) $$\ddot{x}\left(t\right)+\varepsilon \omega \left(\frac{4}{\gamma^{2}}x{\left(t\right)}^{2}-1\right)\ddot{x}\left(t\right)+\omega^{2}x\left(t\right)=0$$

is well-suited for taking these properties into account [11,13]. Here, $\omega =\frac{2\pi }{\tau }$ is the frequency of oscillation (with *τ *describing the circadian period), and *ε *is a flexibility parameter which describes the extent to which the oscillation deviates from the harmonic oscillator model $\ddot{x}\left(t\right)+\omega^{2}x\left(t\right)=0$ describing sinusoidal oscillations with constant phase and amplitude. The parameter *γ *describes a limit cycle amplitude. The nonlinear dynamical system (2) contains a supercritical Hopf bifurcation at the bifurcation point *ε *= 0, which enables limit cycles for positive values of *ε*. The larger the value of *ε*, the faster the limit cycle is approached but the less it resembles a sinusoidal oscillation [15].

Since no exact solution of the van der Pol oscillator is available, we currently use the second-order perturbation solution which is given by

(3) $$x\left(t\right)=a\cos\left(\omega t+\psi \right)-\varepsilon \frac{a{\left(t\right)}^{3}}{8\gamma^{2}}\sin\left(3\omega t+3\psi \right)+O\left(\varepsilon^{2}\right),\;\gamma >0,$$

with the dynamic amplitude function

(4) $$a\left(t\right)=a\left(t;k,\gamma \right)=\frac{1}{\sqrt{\frac{1}{4k}e^{-\varepsilon \omega t}+\frac{1}{\gamma^{2}}}},k\neq 0,\gamma >0,$$

being a solution of the differential equation

$$ȧ\left(t\right)=\varepsilon \frac{a\left(t\right)\omega }{2}\left(1-\frac{a{\left(t\right)}^{2}}{\gamma^{2}}\right)+O\left(\varepsilon^{2}\right)$$

[12]. Here, *k *is a constant of integration and *ψ*_0 _describes an angular phase shift. The initial amplitude *a*_0 _:= *a*(0) depends on *k *and *γ *according to $a_{0}={\left(\frac{1}{4k}+\frac{1}{\gamma^{2}}\right)}^{-\frac{1}{2}}$, showing also dependence of *k *on *a*_0 _and *γ*. The parameters of the circadian component can therefore be summarized by the parameter vector ***β*_0 _**= (*a*_0_, *γ*, *ε*, *ψ*, *τ*).

The linear trend function *c*(*t*) is added to the oscillation in order to enable adaptation of the circadian component to the overall changes in temperature baseline. The van der Pol oscillator describes periodic oscillations with approximately sinusoidal shape that are symmetric around the baseline. Consequently, a decrease in overall amplitude would imply that temperatures decrease about the same amount during the day as they are elevated at night. Under estrogen withdrawal, however, temperature is elevated during nighttime, but remains unmodified at daytime. The linear trend accounts for this one-sided decrease in amplitudes by enabling the actual baseline of the oscillatory component to remain centered between the upper and lower peaks of the sinusoidal waves.

The error terms *e_n _*describe all additional influences on the oscillation, resulting from various causes such as movement, electrical artifacts due to the measurement device or rapid thermoregulatory processes in the tail skin. Different levels of activity during day- and nighttime suggest different variance in these time spans. Even if the variance caused by thermoregulatory processes can be assumed to be small during the quiet phase during the day, husbandry and treatment each morning can lead to excitement periods that largely increase variability of measurements. Furthermore, injection can cause reactions which result in distinct changes in tail skin temperature occurring shortly after the treatment period each morning. In order to cope with these varying scenarios, different variance parameters are assumed for the night period between 6 p.m. and 6 a.m., for the morning period between 6 a.m. and 12 p.m., and for the afternoon period between 12 p.m. and 6 p.m. The random error terms are assumed to be independently and normally distributed, where the distributions are identical within each period. This tributes to the fact that an exact modeling of the 'noise' containing observational errors and treatment effects seems to be very complicated. The assumptions leading to usual least square estimates provide reasonable results as shown below.

The process ${\left(e_{n}\right)}_{n=0}^{N-1}$ is then defined by

$$e_{n}\;\sim \left\{\begin{array}{cc} N\left(0,\sigma_{day1}\right), & n\cdot \Delta t\in T_{day1}\\ N\left(0,\sigma_{day2}\right), & n\cdot \Delta t\in T_{day2},\\ N\left(0,\sigma_{night}\right), & n\cdot \Delta t\in T_{night} \end{array}\right)$$

where *T_day_*_1 _is the period of points in time of 6 - 12 a.m. (distortions due to husbandry and treatment may happen), *T_day_*_2 _is the period between 12 a.m. and 6 p.m. (quiet period, least variance), and *T_night _*is the nighttime period between 6 p.m. and 6 a.m., characterized by increased activity. Thus, model (1) depends on the vector $\beta =\left(\beta_{0},c_{0},c_{1},\sigma_{day1}^{2},\sigma_{day2}^{2},\sigma_{night}^{2}\right)$ containing a total of 10 parameters (note that the 5 parameters of the van der Pol model are comprised in ***β*_0_**).

### Model Fit

A separate model was fitted to the time series obtained from each animal in each experimental phase, aiming at a maximization of the observations' likelihood. Assuming normally distributed and independent error terms *e_n_*, this likelihood is provided by the density function of the multivariate normal distribution:

(5) $$p\left(y_{obs};\hat{\beta }\right)=\frac{1}{{\left(2\pi \right)}^{\frac{N}{2}}|\Sigma \left(\hat{\beta }\right){|}^{\frac{1}{2}}}\exp\left(-\frac{1}{2}{\left(y_{obs}-y\left(\hat{\beta }\right)\right)}^{T}\Sigma {\left(\hat{\beta }\right)}^{-1}\left(y_{obs}-y\left(\hat{\beta }\right)\right)\right).$$

Here, **y***_obs _*is the *N *-dimensional vector of observed measurements, and $y\left(\hat{\beta }\right)$ is the vector of values computed from the estimated model parameters $\hat{\beta }=\left({\hat{\beta }}_{0},{ĉ}_{0},{ĉ}_{1},{\hat{\sigma }}_{day1}^{2},{\hat{\sigma }}_{day2}^{2},{\hat{\sigma }}_{night}^{2}\right)$, with ${\hat{\beta }}_{0}=\left({\hat{a}}_{0},\hat{\gamma },\hat{\varepsilon },\hat{\psi },\hat{\tau }\right)$.

The covariance matrix $\Sigma \left(\hat{\beta }\right)$ contains the estimated individual variances for one of the three previously defined time periods on its diagonal, and all of its off-diagonal entries are zero.

Equation (5) can be reformulated to the negative log-likelihood, which serves as the objective function for optimization:

(6) $$-l\left(y_{obs};\hat{\beta }\right)=\frac{N}{2}\log\left(2\pi \right)+\frac{1}{2}\log\left(\left|\Sigma \left(\hat{\beta }\right)\right|\right)+\frac{1}{2}{\left(y_{obs}-y\left(\hat{\beta }\right)\right)}^{T}\Sigma {\left(\hat{\beta }\right)}^{-1}\left(y_{obs}-y\left(\hat{\beta }\right)\right).$$

In order to improve performance and runtime of the optimization, model fit was performed by pseudo-maximum likelihood estimation. In this context, a subset of the model parameters was estimated prior to each step of the optimization iteration. In a first step, the linear trend parameters (mean *c*_0 _and slope *c*_1_) were estimated by linear regression for each phase. These values stayed fixed in the subsequent optimization. Variance parameters for the residuals were derived from the empirical variances of the residuals in each step of the iteration.

The period of oscillation was restricted to $\hat{\tau }=24$ hours to account for the fixed light-dark cycle to which the animals were subjected.

Optimization was then performed only for the remaining 4 parameters contained in ***β*_0_**. Therefore, instead of searching for a global minimum of the 10-dimensional negative log-likelihood function, the minimum of this function with respect to a subset of these parameters was assessed, while the other parameters were fixed. Optimization was performed in R, using the BFGS quasi-Newton method with box constraints [16].

Initial values for start amplitude *a*_0 _were obtained by fitting a harmonic regression model *y*(*t*) = *A*_1 _cos(*ωt*) + *A*_2 _sin(*ωt*) to the observations of the first day in each experimental phase, as described in the literature [10] (with experimental phases being denoted as P_1 _- P_3 _as described in the *The Experiments *in the *Methods *section). Starting values were then given by $a_{0}=\sqrt{A_{1}^{2}+A_{2}^{2}}$. Starting values for the limit cycle amplitude *γ *were obtained in similar manner from the last day of each experimental phase. Since aftereffects of surgery can prevent regular rhythms at day one of measurements, but surgery-induced hypothermia can lead to unrealistically large estimates of *a*_0_, small start amplitudes were promoted by starting optimization with initial value *a*_0 _= 0.5 in P_1_. Upper boundaries for parameter *a*_0 _(or *γ*) were computed as half of the maximum span of values occurring during the first (or last) day in each phase.

In contrast to previous studies where limit cycle amplitudes had been determined prior to the start of the actual experiment for each individual [12], here, the true limit cycle amplitudes were unknown and had to be estimated together with the other model parameters simultaneously. To ensure realistic estimates for *γ*, we had to bound *ε *from below. Otherwise, extremely large estimates for *γ *could occur, which were then compensated by nearly vanishing values of *ε*. To this end, we restricted *ε *to a lower boundary of 0.2. In those cases where no dynamical effects on the amplitudes occurred, i.e. due to unsuccessful treatment in P_3_, estimates of start and limit cycle amplitude were roughly similar. Consequently, the lower boundary on *ε *could not artificially induce amplitude growth in those cases. The upper boundary for *ε *was set to 1 in order to restrict the deviation of the perturbation solution from the true van der Pol oscillator to small values. The phase shift *ψ *was constrained to the interval [-*π*,*π*].

When subjecting the initial values of each parameter to small normally distributed perturbations of variance 0.1, the average absolute differences between the corresponding parameter estimates and the estimates obtained using unperturbed initial values lay between 6.2 · 10^-6 ^(parameter *ε*) and 2.5 · 10^-4 ^(parameter *a*_0_). This indicates that (1) the large fluctuations in the data apparently prevent the optimization routine from finding the global minimum, but (2) that nevertheless, the model fit should be sufficiently robust against small alterations in the initial values, so that changes in starting value do not drastically influence the result of the fit.

## Results and discussion

### Exemplary illustration of the model fit

**Figure 4:**
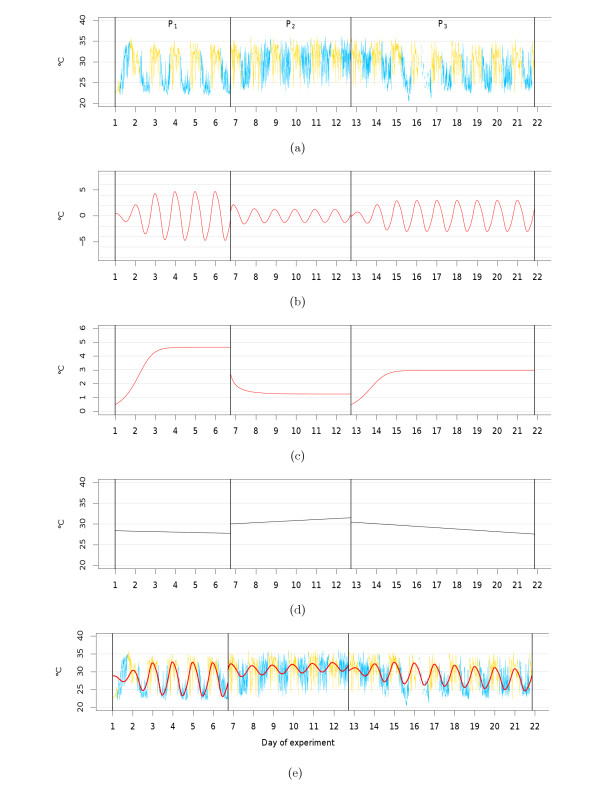
**Example of model fit (E2 group)**. Examples of estimated model components belonging to an animal of the *E2 *group, separated by experimental phase (P_1 _- P_3_). (a) Tail skin temperature time measurements (*y_n_*); (b) circadian components $\left(\hat{x}\left(t\right)\right)$; (c) amplitude functions $\left(\hat{a}\left(t\right)\right)$; (d) trend curves (ĉ(t)); (e) combination of estimated circadian components and trend curves $\left(\hat{x}\left(t\right)+ĉ\left(t\right)\right)$, embedded in the time series. Daytime periods are shown in yellow; nighttime periods are shown in blue. The model parameter estimates are given in the Tables 1 and 2.

**Figure 5:**
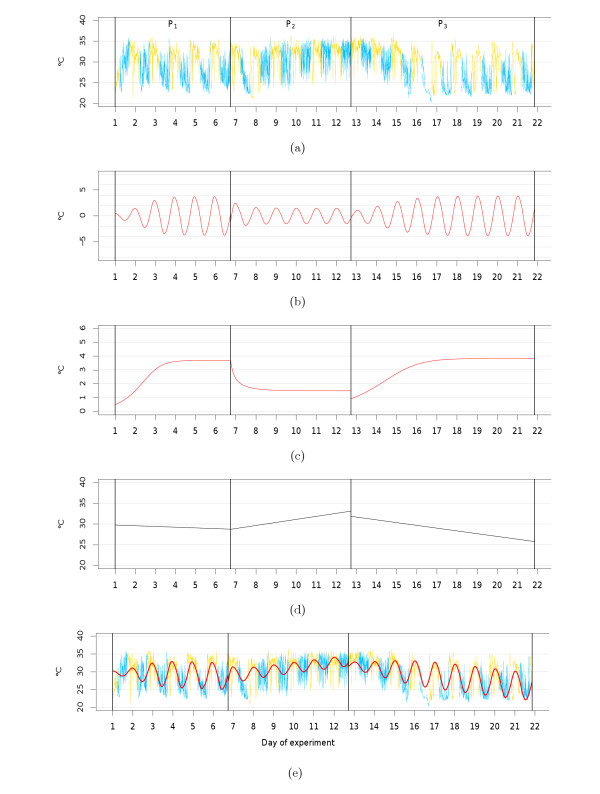
**Example of model fit (Tibolone group)**. Examples of estimated model components belonging to an animal of the *Tibolone *group, separated by experimental phase (P_1 _to P_3_). (a) Tail skin temperature time measurements (*y_n_*); (b) circadian components $\left(\hat{x}\left(t\right)\right)$; (c) amplitude functions $\left(\hat{a}\left(t\right)\right)$; (d) trend curves (ĉ(t)); (e) combination of estimated circadian components and trend curves $\left(\hat{x}\left(t\right)+ĉ\left(t\right)\right)$, embedded in the time series. Daytime periods are shown in yellow; nighttime periods are shown in blue. The model parameter estimates are given in the Tables 1 and 2.

Figures 4 and 5 show examples of the circadian components *x*(*t*), the corresponding amplitude functions *a*(*t*) and the linear trend components detected in the tail skin temperature time series of two exemplary animals. The parameter estimates obtained for each animal are provided in Table 1 (van der Pol parameters) and in Table 2 (trend and variance parameters). Note that the residuals obtained for the considered time periods are unimodally distributed with a slight difference to a normal distribution. However, analysis of the autocorrelation function revealed only very short-time dependencies in the residuals. This is not in complete accordance with our assumptions, but does not contradict the point that our model serves as a rough description of the considered processes.

**Table 1:** Examples of estimated van der Pol parameters.

Examples of estimated van der Pol parameters										
			***E2***					***Tibolone***		
	$\hat{a}\left(0\right)$	$\hat{\varepsilon }$	$\hat{\psi }$	$\hat{\gamma }$	$\hat{a}\left(T\right)$	$\hat{a}\left(0\right)$	$\hat{\varepsilon }$	$\hat{\psi }$	$\hat{\gamma }$	$\hat{a}\left(T\right)$

P_1_	0.5000	0.4969	0.0100	4.6416	4.6416	0.5000	0.3753	0.2452	3.6932	3.6931
P_2_	2.7760	0.2000	0.4055	1.2618	1.2620	3.8264	0.2000	-0.1275	1.4968	1.4971
P_3_	0.5000	0.4552	-0.1423	2.9695	2.9695	0.9150	0.2000	-0.2483	3.8367	3.8363

Examples of van der Pol model parameters estimated for animals of the *E2 *group and the *Tibolone *group. The estimated circadian components and amplitude functions are illustrated in Figures 4 and 5. $\hat{a}\left(0\right)$: initial amplitude, $\hat{\varepsilon }$: flexibility parameter, $\hat{\psi }$: angular phase shift, $\hat{\gamma }$: limit cycle amplitude, $\hat{a}\left(T\right)$: last amplitude function value estimated for an experimental phase.

**Table 2:** Examples of estimated trend and variance parameters

Examples of estimated trend and variance parameters										
***E2***						***Tibolone***				
	${ĉ}_{0}$	${ĉ}_{1}$	${\hat{\sigma }}_{day1}^{2}$	${\hat{\sigma }}_{day2}^{2}$	${\hat{\sigma }}_{night}^{2}$	${ĉ}_{0}$	${ĉ}_{1}$	${\hat{\sigma }}_{day1}^{2}$	${\hat{\sigma }}_{day2}^{2}$	${\hat{\sigma }}_{night}^{2}$

P_1_	28.3782	-0.0001	11.0133	3.9545	5.0698	29.7567	-0.0001	8.9852	3.4665	7.7807
P_2_	30.0259	0.0002	7.2154	4.4644	8.1160	28.7178	0.0005	12.5643	4.2927	8.5226
P_3_	30.4638	-0.0002	9.3479	3.9833	6.6188	31.8452	-0.0005	17.5731	3.4213	7.9173

Examples of trend and variance parameters estimated for animals of the *E2 *group and the *Tibolone *group. The corresponding time series and the estimated trend functions are illustrated in Figures 4 and 5. ${ĉ}_{0}$: intercept, ${ĉ}_{1}$: slope, ${\hat{\sigma }}_{day1}^{2}$: variance of first daytime period (6 - 12 a.m.), ${\hat{\sigma }}_{day2}^{2}$: variance of second daytime period (12 a.m. - 6 p.m.), ${\hat{\sigma }}_{night}^{2}$: variance of nighttime period (6 p.m. - 6 a.m.).

In both animals, oscillations vanish after surgery, with the limit cycle being approached after day two (*E2 *group animal) or day three (*Tibolone *group animal) in P_1_. (For this and the following, compare (a), (b) and (c) in Figures 4 and 5) Amplitudes quickly decrease to a new constant level in P_2 _and rise again after substance treatment in P_3_. In each phase, limit cycle amplitudes *γ *closely coincide with the final values of the estimated amplitude function (*a*(*T*)), indicating that the limit cycles amplitude provides a good representation of the amplitude at the end of experiments. For both animals, limit cycle amplitudes approximately double from P_2 _to P_3_, indicating positive effects of each substance on the extent of circadian oscillation.

The reduction in the decrease of nighttime temperatures in P_2_, as well as its subsequent restoration in P_3 _are also reflected by positive or negative trends in these different experimental phases, respectively.

In total we fitted the three phases for 20 animals, with AIC values between 12000 and 23000, each being slightly lower than the value obtained for the corresponding fit of the harmonic regression model. Average parameter estimates obtained for both treatment groups are provided in Table 3 and 4.

**Table 3:** Estimated van der Pol parameters

Estimated van der Pol parameters						
**Group**	**Experimental phase**	$\hat{a}\left(0\right)$	$\hat{\varepsilon }$	$\hat{\psi }$	$\hat{\gamma }$	$\hat{a}\left(T\right)$

*E2*	P_1_	0.65 ± 0.32	0.52 ± 0.26	0.24 ± 0.19	3.97 ± 1.48	3.92 ± 1.40
	P_2_	2.48 ± 0.98	0.29 ± 0.25	0.40 ± 0.34	1.58 ± 0.55	1.58 ± 0.55
	P_3_	1.43 ± 0.61	0.31 ± 0.26	0.16 ± 0.36	2.70 ± 1.00	2.70 ± 1.00

*Tibolone*	P_1_	0.57 ± 0.21	0.56 ± 0.34	0.25 ± 0.28	3.55 ± 0.75	3.52 ± 0.71
	P_2_	3.10 ± 1.37	0.36 ± 0.34	0.34 ± 0.37	1.61 ± 0.55	1.61 ± 0.55
	P_3_	1.45 ± 0.67	0.20 ± 0.00	0.05 ± 0.21	3.29 ± 0.99	3.29 ± 0.99

Mean values and standard deviations of the van der Pol model parameters estimated for the *E2 *and the *Tibolone *group (sample size: 10 animals per group). $\hat{a}\left(0\right)$: initial amplitude, $\hat{\varepsilon }$: flexibility parameter, $\hat{\psi }$: angular phase shift, $\hat{\gamma }$: limit cycle amplitude, $\hat{a}\left(T\right)$: last amplitude function value estimated for an experimental phase.

**Table 4:** Estimated trend and variance parameters

Estimated estimated trend and variance parameters						
**Group**	**Experimental phase**	${ĉ}_{0}$	${ĉ}_{1}$	${\hat{\sigma }}_{day1}^{2}$	${\hat{\sigma }}_{day2}^{2}$	${\hat{\sigma }}_{night}^{2}$

*E2*	P_1_	29.53 ± 1.68	(0.31 ± 1.99) · 10^-4^	9.87 ± 3.69	4.06 ± 1.28	10.39 ± 2.76
	P_2_	30.35 ± 1.12	(1.37 ± 1.39) · 10^-4^	9.56 ± 3.25	4.91 ± 3.55	11.17 ± 2.40
	P_3_	30.71 ± 0.74	(-1.46 ± 0.62) · 10^-4^	10.22 ± 3.71	3.55 ± 0.90	9.75 ± 2.07

*Tibolone*	P_1_	30.02 ± 1.01	(1.14 ± 2.85) · 10^-4^	9.83 ± 2.78	5.25 ± 1.79	15.07 ± 5.24
	P_2_	30.23 ± 1.86	(1.53 ± 2.58) · 10^-4^	10.78 ± 2.51	5.85 ± 1.33	11.97 ± 2.74
	P_3_	30.86 ± 1.28	(-3.15 ± 1.15) · 10^-4^	14.77 ± 3.50	4.57 ± 1.34	11.28 ± 2.68

Mean values and standard deviations of the trend and variance parameters estimated for the *E2 *and the *Tibolone *group (sample size: 10 animals per group). ${ĉ}_{0}$: intercept, ${ĉ}_{1}$: slope, ${\hat{\sigma }}_{day1}^{2}$: variance of first daytime period (6-12 a.m.), ${\hat{\sigma }}_{day2}^{2}$: variance of second daytime period (12 a.m.- 6 p.m.), ${\hat{\sigma }}_{night}^{2}$: variance of nighttime period (6 p.m. - 6 a.m.).

### Effects of the treatment in P_3 _on the amplitude parameters

After successful treatment, we expect amplitudes to increase from P_2 _to P_3_. When searching for treatment effects, we thus focus on the limit cycle amplitudes parameter *γ *which indicates the state of the system at the end of these experimental phases.

**Figure 6:**
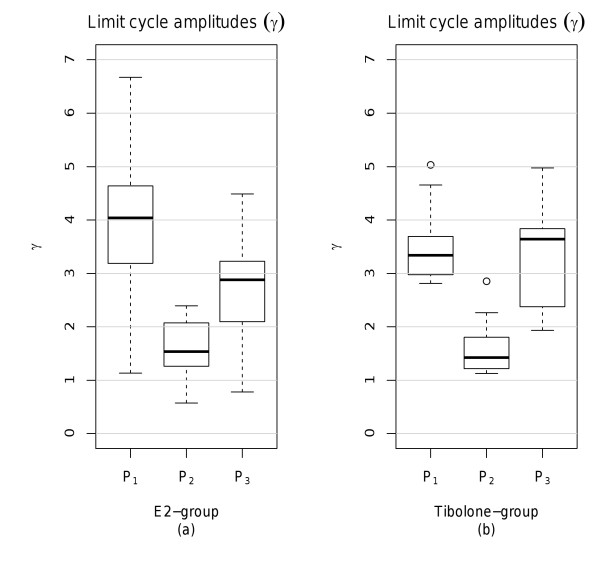
**Estimated limit cycle amplitudes per phase and treatment group**. Limit cycle amplitudes occurring at the end of each experimental phase. (a) *E2 *group, (b) *Tibolone *group.

Boxplots of the limit cycle amplitudes obtained from the time series of each treatment group (10 animals per group) are shown in Figure 6. As expected, amplitudes at the end of P_2 _tend to be located at lower levels than those at the end of P_1_. In P_3_, an overall rise in amplitude parameters can be observed relative to P_2_.

**Figure 7:**
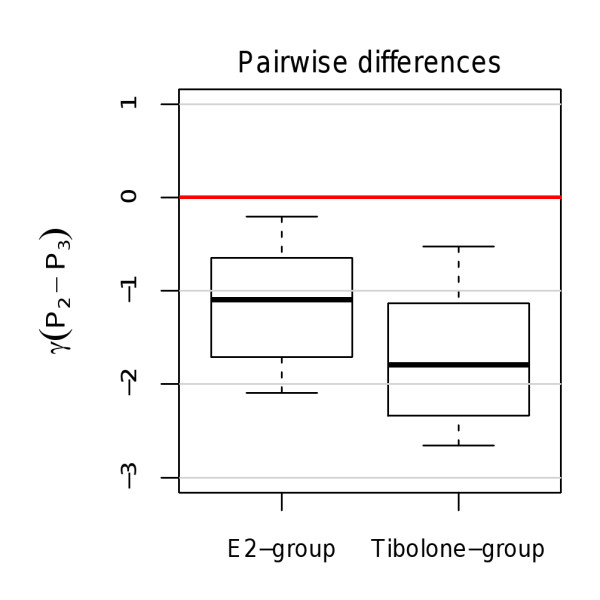
**Changes in estimated amplitudes between vehicle and treatment phase**. Pairwise differences (P_2 _- P_3_) of limit cycle amplitudes estimated in both treatment groups.

In order to illustrate amplitude reconstruction during treatment in P_3_, Figure 7 provides boxplots of the pairwise differences in limit cycle amplitudes of vehicle and treatment phase (P_2 _- P_3_). As expected, all pairwise difference values are located below zero, indicating increase in amplitudes from P_2 _to P_3 _and thus successful amplitude restoration in both groups.

In order to assess whether this increase is statistically significant and to obtain quantitative estimates of the amount of amplitude reconstruction in each treatment group, Table 5 provides mean estimates and confidence intervals of the pairwise differences. To account for the uncertainty of the parameters' distributions, the estimates and their confidence intervals have been computed by bootstrapping of the mean with 1000 bootstrap samples being drawn in each simulation [17]. All confidence intervals in Table 5 are located below zero, indicating a significant increase in amplitudes from P_2 _to P_3 _in both treatment groups. In the *E2*-group, an average amplitude rise of approximately 1.13°C occured. Amplitude rise due to *Tibolone *amounted to an average of 1.68°C.

**Table 5:** Mean differences between vehicle and treatment phase

Mean differences between vehicle and treatment phase		
**Treatment group**	$\left(\hat{\mu }\right)$	**CI**

*E2*	-1.1265	(- ∞, - 0.8247)
*Tibolone*	-1.6807	(- ∞, - 1.3224)

Mean estimates $\left(\hat{\mu }\right)$ and one-sided 95%-bootstrap confidence intervals (CI) of the pairwise differences (P_2 _- P_3_) in limit cycle amplitudes *γ *after treatment with *E2 *and *Tibolone *in P_3_.

### Comparing amplitude reconstruction between the E2 and the Tibolone group

In order to compare the performances of both treatments, we computed the ratios $Q^{\left(k\right)}:=\frac{\gamma_{3}^{\left(k\right)}}{\gamma_{1}^{\left(k\right)}}$ of limit cycle amplitudes between P_3 _and P_1 _for all animals *i *= 1, ..., 10 of each group *k *(*k *either refers to *E2 *or*Tibolone*). These ratios provided a measure for the proportion of the natural amplitude values, as observed at the end of P_1_, that have been reconstructed during treatment in P_3_.

We compared the ratios obtained for each group by a one-sided bootstrap test of the median for independent samples (1000 bootstrap samples, significance level *α *= 0.05). The corresponding hypothesis pair was

(7) $${\text{H}}_{\text{0}}:\theta^{\left(E2\right)}-\theta^{\left(TI\right)}\geq 0.2,\;\;{\text{H}}_{\text{1}}:\theta^{\left(E2\right)}-\theta^{\left(TI\right)}<0.2,$$

where *θ*^(*E2*) ^and *θ *^(*TI*) ^are the location parameters of the ratio distributions in the *E2 *or *Tibolone *group, respectively. As shown in Table 6, the upper confidence interval boundary is slightly above zero, but far below the threshold of 0.2. This indicates that the *Tibolone *group exhibits a similar recovery rate in P_3 _as the *E2 *group.

**Table 6:** Differences between the amounts of amplitude reconstruction in the E2 and the Tibolone group

Differences between the amounts of amplitude reconstruction in the *E2 *and the *Tibolone *group			
**Estimator**	$\left(\hat{\mu }\right)$	**CI**	***p*_0.2_**

*θ*^(*E2*) ^- *θ *^(*TI*)^	-0.2294	(- ∞, 0.0078)	0.0031

Median estimates $\left(\hat{\mu }\right)$ and one-sided 95% - bootstrap confidence intervals (CI) of the differences between the limit cycle amplitudes ratios obtained in both treatment groups. The p-value (*p*_0.2_) was obtained by applying the hypothesis pair in equation (7) to test whether this difference was below the threshold 0.2.

## Conclusions

Usually, telemetric data analysis is based on simple graphical methods to illustrate time series, with compounds being assessed subjectively afterwards. Sometimes, the information within the huge amount of longitudinal measurements is condensed to a few mean values and then, statistical inference is performed by using simple methods like a t-test to compare groups. In doing so, mean temperatures are calculated, for example, for successive 30 minute intervals [5], entire nighttime periods [6] or time spans of several hours after substance injection [4,8].

Here, we propose a new statistical strategy to analyze stochastic dynamic data in order to detect promising compounds for hot flush treatment using the whole time series. Our methodology is based on a nonlinear dynamical system defined by the van der Pol equation. The resulting differential equation model consists of sensible and well-interpretable model parameters to fit and to analyse tail skin temperature measurements including circadian rhythms. The mechanism presented here is a modified version of the models for human body core temperature series [11-13]. As an improvement to these models, an overall trend in temperature baseline has been included, and, most importantly, separate day- and nighttime variances are used to account for varying levels of activity and excitement at different times of the day. Moreover, common approaches model the circadian component by sinusoidal oscillations with constant amplitudes (i.e. Cosinor and harmonic regression models [10]). The fundamental advantage of our methodology is to involve a dynamic amplitude function which captures the time-dependent changes in amplitude during the treatment phase.

Two ideas for slight improvements for future work are suggested in the following. The first is related to the fact that the model fit was performed by pseudo-maximum likelihood fitting based on the assumption of independent residuals. This assumption does not represent true characteristics of the residual data, since high frequency oscillations are mainly based on short-term thermoregulatory processes, and thus measurements are correlated. Consequently, knowledge of short-term dependencies could provide more realistic models. To this end, the use of ARMA (auto-regressive moving average) processes has been suggested to model the thermoregulatory component in body core temperature [11].

Secondly, substance effects have only been investigated with respect to their implications on amplitude parameters. However, other van der Pol model parameters exist which describe different characteristics of circadian rhythms, e.g. the amplitude flexibility *ε*, the phase shift *ψ *or the differences between start amplitudes and final amplitudes of a certain experimental phase. The influence of different treatment modes on these parameters have to be investigated. It could, for example, be examined whether significant differences in these types of parameters occur in the *E2 *group. Observed effects could then be compared to those effects probably occurring in test groups of various substances.

## Competing interests

The authors declare that they have no competing interests.

## Authors' contributions

The work presented here is based on a collaboration between all authors working extensively on the experiments described in this paper. KP and VP designed and supervised the experiments. DG, BWI, KK and RV provided the mathematical and statistical framework and developed analytical tools. DG did the programming and the implementation. DG and BWI analyzed the data and DG wrote the main parts of the paper. All authors discussed the results and implications and commented on the manuscript at all stages. All authors read and approved the final manuscript.
